# Burden and Depression among Caregivers of Visually Impaired Patients in a Canadian Population

**DOI:** 10.1155/2016/4683427

**Published:** 2016-03-08

**Authors:** Zainab Khan, Puneet S. Braich, Karim Rahim, Jaspreet S. Rayat, Lin Xing, Munir Iqbal, Karim Mohamed, Sanjay Sharma, David Almeida

**Affiliations:** ^1^Department of Ophthalmology, Hotel Dieu Hospital, Queen's University, 166 Brock Street, Kingston, ON, Canada K7L 5G2; ^2^Department of Ophthalmology, Virginia Commonwealth University School of Medicine, 401 N. 11th Street, Suite 439, Richmond, VA 23219, USA; ^3^VitreoRetinal Surgery, PA, 7760 France Avenue S., Minneapolis, MN 55435, USA

## Abstract

*Purpose/Background*. This study reports the degree of burden and the proportion at risk for depression among individuals who provide care to visually impaired patients.* Study Design*. This is clinic-based, cross-sectional survey in a tertiary care hospital.* Methods*. Caregivers were considered unpaid family members for patients whose sole impairment was visual. Patients were stratified by vision in their better seeing eye into two groups: Group 1 had visual acuity between 6/18 and 6/60 and Group 2 were those who had 6/60 or worse. Burden was evaluated by the Burden Index of Caregivers and the prevalence of being at risk for depression was determined by the Center for Epidemiologic Studies Depression scale.* Results*. 236 caregivers of 236 patients were included. Total mean BIC scores were higher in Group 2. Female caregivers, caregivers providing greater hours of care, and caregivers of patients who have not completed vision rehabilitation programs are at higher risk for depression.

## 1. Introduction

Burden of care has been defined as the financial physical, psychological, and social discomfort experienced by the principal caregiver of a disabled family member [1]. Care burden has been reported to significantly increase the risk for mortality among caregivers of elderly spouses with at least moderate disability irrespective of etiology [2]. Moreover, depression among caregivers is higher during the period at which they provide care, and burden is positively correlated with depression within this time frame [3].

The majority of the literature on this topic comes from the evaluation of caregivers of patients with intractable neurological diseases (e.g., Alzheimer's and Parkinson disease) [4]. However, recent studies have examined the role of burden in the caregivers of patients with cancer [5], eating disorders [6], and lung transplants [7]. In the ophthalmic literature, prior studies have examined the prevalence of depression and diminished quality of life reported by blind patients themselves [8, 9]. Recently, however, there have been investigations [10] on the quantitative evaluation of burden and depression faced by caregivers of individuals with visual impairment in India and the USA. This study examines this relationship in a Canadian population.

In North America, unipolar depressive disorders are the 2nd leading cause of disability-adjusted life years (DALY) and the 4th cause of DALY worldwide [11]. In Canada alone, it is estimated that 8% of the general adult population will experience a major depressive episode at some point in their lives [12]. Mood disorders such as depression have major economic impact through associated health care costs as well as lost work productivity. According to the Public Health Agency of Canada [13], this impact is dual in nature. Firstly, it comes with the associated loss of productivity in the workplace due to absenteeism and diminished effectiveness. Secondly, it comes with the high health care costs attributable to primary care visits, hospitalizations, and medication. Another aspect of mental health is the burden experienced by individual caregivers which may not directly affect health care costs but is still compelling. A recently published study examining the psychological distress of caregiving and noncaregiving twins found that caregiving was associated with distress as measured by mental health functioning, anxiety, perceived stress, and depression [14]. Bernbaum et al. [15] found that diabetes-related visual impairment was a major stressor in marital relationships. In their sample of either legally blind or no light perception (NLP) patients, 50% were separated or divorced within a mean of 1.6 years of the onset of visual impairment regardless of the length of the relationship prior to vision loss. The risk of separation or divorce was comparatively higher in couples where one partner had no light perception (NLP) vision. A longitudinal study done by Strawbridge et al. [16] found that spouses of patients with vision loss had an increased risk of poorer physical and emotion well-being over 5 years. Researchers postulate that visual impairment results in the loss of unseen gestures and body language thus having an effect on communication between partners [17].

Severe visual impairment is known to impact the social and economic prosperity of the patient, the family, and community in which they reside [18–20]. As the proportion of older adults in Canada rises, the number of Canadians with age related ocular disease and vision impairment is predicted to increase substantially within the next few decades [21, 22]. In light of these facts and the growing attention of public health issues surrounding the burden of disease, we conducted a cross-sectional study to measure the care burden and the proportion of those at risk for depression among caregivers of legally blind and low vision patients in a population receiving care in Kingston, Ontario, Canada. Undoubtedly, caregivers of all patients, regardless of age, with severe visual impairments can presumably experience substantial care burden. Nonetheless, the scope of this study was to assess adult patients from a clinic that is predominantly comprised of patients with advanced macular degeneration and diabetic retinopathy. Furthermore, while there is overlap between depressive symptomatology and depressive disorders, the aim of this study in assessing patients at risk of depression is basing this risk upon the depressive symptomatology. Making a formal diagnosis of depression is beyond the scope of what could be accomplished through questionnaires and would require the aid of psychiatrists. The aims of this study were to (1) report and contrast the burden faced by caregivers of patients who either are legally blind or have low vision, (2) explore factors related to burden faced by caregivers, (3) elucidate the proportion of those at risk for depression among caregivers of these patients, and (4) explore factors related to being at risk for depression in these caregivers.

## 2. Methods

### 2.1. Study Design

This study is clinic-based, cross-sectional survey of caregivers of patients with visual impairment.

### 2.2. Participants and Procedures

This study was conducted in accordance with the tenets of the Declaration of Helsinki and all provincial and federal laws. It received approval from the Queen's University (Kingston, Ontario, Canada) research ethics board committee. All participants provided informed consent; this included caregivers as well as patients. When possible, consent forms were signed in person. However, many participants served by the eye clinic travelled from large distances and required phone consent if they were unable to appear in person to sign the appropriate paperwork.

Patients were divided into 2 groups based on their severity of visual impairment: Group 1 had visual acuity better than 6/60 yet still worse than 6/18 (low vision) and Group 2 were those with 6/60 or worse (legally blind). Participants were recruited from the medical retina clinics at one institution (Hotel Dieu Hospital) in Kingston, Ontario, in Canada. While no specific disease related exclusion criteria were outlined, all patients in our clinic had either a diagnosis of diabetic retinopathy secondary to Type 2 diabetes or age related macular degeneration. We included caregivers of all patients with visual impairment requiring aide from a caregiver with either their activities of daily living (ADL) or instrumental activities of daily living (IADL). Caregivers were eligible if they were a family member or a friend that the blind patient identified as the “person they usually turn to for help regarding their care.” Caregivers had to be adults who were unpaid for their support, able to converse in English, and provided care at the patients' homes (nursing home and assisted living center patients were excluded). Adults were defined as being over the age of 18 years. All patients or caregivers who were unable to give informed consent were excluded from this study. Caregivers were interviewed by telephone or in person using validated multidimensional instruments. The study period was from September 2011 to April 2012. The formal exclusion criteria consisted of the patient having intractable neurological disease, physical handicap, mental handicap, prior stroke, renal dialysis, cancer, dementia, severe motor deficits, or any condition which rendered the patient unable to ambulate. Examples of patient comorbidities that were not excluded were hypertension, diabetes mellitus, hyperlipidemia, mild inflammatory arthritis, mild degenerative joint disease, osteoporosis, mild to moderate chronic obstructive pulmonary disease (COPD), hearing impairment, obesity (excluding morbid obesity), and congestive heart failure New York Heart Association (NYHA) class I or II (excluding classes III and IV). This criterion was directed to isolate those caregivers that needed to provide care predominantly due to a patient's visual impairment. The same exclusion criteria applied to caregivers.

### 2.3. Measurements

#### 2.3.1. Burden Index of Caregivers (BIC)

This is a multidimensional scale that measures care burden and has been previously validated [23]. It is composed of five burden domains: (1) time-dependent, (2) emotional, (3) existential, (4) physical, and (5) service-related burden. Each domain consists of 2 questions with each question assessed using a 5-point Likert scale (0 = never, 1 = almost never, 2 = sometimes, 3 = often, and 4 = always). There is one additional item that assesses overall burden, that is, “How burdensome do you think providing care is to you?” In total, there are 11 questions (Appendix A).

#### 2.3.2. Center for Epidemiologic Studies Depression (CES-D) Scale

The CES-D scale was developed by the United States' National Institute of Mental Health. This is a scale of 20 questions used to identify individuals at risk for depression [20]. Responses indicate the number of days per week the subject was affected by depressive symptoms (0 days with a score of 0; 1-2 days with a score of 1; 3-4 days with a score of 2; and >5 days with a score of 3). Scores can range from 0 to 60, with a higher score representing a stronger tendency toward depression. As done in multiple prior studies [11, 24–26], a score ≥16 was used to indicate those at risk for major depression (Appendix B).

### 2.4. Statistical Analysis

Mean scores of total BIC and personal estimates of overall burden were calculated for the two groups. Independent two-sided *t*-tests were used with *α* = 0.05 to compare the mean BIC scores between the two groups. The mean scores for the total BIC measure and the personal estimate of overall burden were the dependent variables for the linear regressions models used to determine which factors significantly contributed to caregiver burden. Independent variables were the numerous participant characteristics (see Table 1). With these parameters defined, a backwards selection technique was used.

A *Z*-test was used to compare the two groups regarding the proportion of patients at risk for depression. To determine the covariates associated with the risk of depression, we used the demographic data (Table 1) as the independent variables for our logistic regression model. The binary dependent variable was risk of depression (yes or no, based on a CES-D score of ≥16). Backwards selection was used to exclude any variables that did not contribute significantly to the fit of the model. All statistical analysis was done with SAS 9.3 (2011 SAS Institute Inc., Cary, NC, USA).

## 3. Results

### 3.1. Participant Characteristics

A total of 236 caregivers completed the survey, all of which were viable for the final analysis. Of the 236 participating caregivers, 160 were from Group 1 (low vision without being legally blind) and 76 were from Group 2 (legally blind). Table 1 shows demographic and other information collected from participants. The mean age of patients and caregivers was 76.4 and 64.8, respectively. The majority of patients and caregivers were female, 54% and 59%, respectively. The majority of the caregivers were either adult children (30%) or spouses (52%). The proportion of patients and caregivers with at least one chronic illness was 48% and 26%, respectively. Nearly three-quarters of the patients were visually impaired due to age related macular degeneration (ARMD) or its sequelae. Moreover, roughly three-quarters of the patients also completed vision rehabilitation at least once in their lifetime.

### 3.2. Care Burden: Low Vision versus Legally Blind Patients

Mean scores for each of the BIC measures were stratified by group (Table 2). The BIC scores were significantly higher for Group 2 compared to Group 1 for all of the domains as well as the personal estimate of overall burden and the total BIC (*P* < 0.01). Amongst the individual domains, the greatest difference between the groups was seen in time-dependent burden followed by the emotional burden. The smallest difference was noted for service-related burden.

### 3.3. Covariates Impacting the Fit of the 2 Linear Regression Models

Covariates significantly impacting the fit of the 2 models are shown in Table 3. Daily hours of close supervision were significant for both measures. Examples of close supervision consisted of bathing the patient, grooming the patient, acting as a walking guide, transferring and transporting patient, and so forth. Patients who had not completed a vision rehabilitation program at least once during their lifetime were shown to have caregivers with higher burden scores. The participant characteristics that were not significant in either of these measures were visual acuity, age, relationship to the patient, duration of caregiving years, etiology of vision loss, presence of chronic illness, and the number of supplemental caregivers.

### 3.4. Prevalence of Caregivers at Risk for Depression

**Figure 1:**
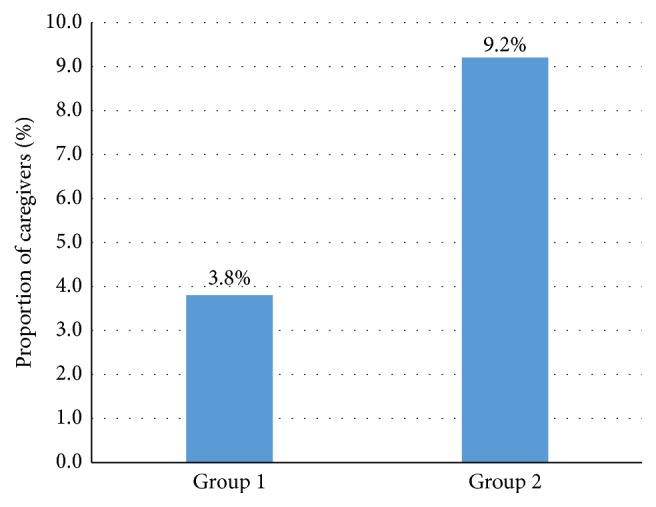
Proportion of caregivers at risk for depression (by Center for Epidemiologic Studies Depression [CES-D] scale). Group 1: best corrected visual acuity in the better eye >6/60 yet worse than 6/18. Group 2: best corrected visual acuity in the better eye ≤6/60 (legally blind).

Figure 1 illustrates the prevalence of those at risk for depression, reflected by a CES-D score ≥16. The proportion of caregivers at risk of depression increased from Group 1 to Group 2, although this difference was not statistically significant (*P* = 0.11). Group 2 had 7 caregivers at risk for depression (9.2%) and Group 1 had 6 at risk caregivers (3.8%).

### 3.5. Factors Related to the Risk of Depression among Caregivers

The covariates significantly correlated to depression risk are shown in Table 4. Caregivers providing close supervision for ≥2.5 hours per day were at 7.45 increased odds of depression compared to those who provided <2.5 hours. Female caregivers compared to male caregivers had roughly fivefold higher odds of being at risk for depression. Caregivers of patients who did not complete at least one vision rehabilitation program in their lifetime were at 4.23 increased odds of being at risk for depression compared to caregivers of patients who completed at least one vision rehabilitation program.

Caregivers identified as being at risk for depression were contacted by the principal investigator and questioned about suicidal ideation or thoughts of harming oneself. Fortunately, none of the caregivers in this study endorsed such thoughts. However, they were all encouraged to seek an evaluation with a mental health provider or their primary care physician. An appointment was offered at the local university hospitals where the study was conducted.

## 4. Discussion

The investigation of burden and depression among caregivers has been a popular area of study for patients with disabilities secondary to neurological diseases. The study of this relationship among caregivers of visually impaired patients is an emerging field in public health and an important one since caregivers play a vital role in the well-being of patients. Instrumental assistance (e.g., providing transportation, managing finances) from family members is associated with better adaption to vision loss, fewer depressive symptoms, and greater life satisfaction [27–30].

Previous studies reported the burden and depression risk amongst caregivers in India and the USA. However, a Canadian population has not been evaluated to the same degree. The differences between health care structures, cultures, and patient demographics between these populations have generated an interest in undertaking this study in Canada.

The mean burden scores for each of the 5 domains revealed that individuals providing care to patients who are legally blind experience higher burden than those providing care to patients who are not legally blind but meet the threshold for low vision. We speculate that caregivers of patients with low vision do not experience as much burden because these patients still retain enough vision which can be improved by low vision aids (e.g., magnifying glasses and closed circuit television systems). Perhaps this results in greater independent functioning and a decreased reliance on caregivers. Furthermore, the burden scores for caregivers of the patients with low vision were considerably lower than the caregivers of legally blind patients from studies performed in India and the USA. It appears that being legally blind imparts considerable limitations on an individual compared to having low vision. For example, in the province of Ontario, certain kinds of low vision patients may still be allowed to operate noncommercial motor vehicles, albeit with restrictions, whereas a legally blind patient is not permitted to operate motor vehicles at all. Instances like these are just one example of how a legally blind patient has greater limitations compared to someone with low vision. It is plausible to see how legally blind patients would have some degree of reduction in their personal freedom and capabilities thus increasing their reliance on family members for simple needs. The greatest disparity among the various domain scores was for the time domain burden, revealing that the perceived burden related by the caregivers was due to limitations on their personal time. Given that the maximum score for each domain could be 4, the low scores for the remaining domains imply that caregivers of patients with visual impairment perceive relatively small amounts of burden. Another contributor to the low burden scores could be the widespread use of vision rehabilitation amongst our sample of patients. Vision rehabilitation and low vision programs were completed by 72% of our sample at least once in their lifetime. It is conceivable that skills learned during these programs enable patients with low vision to be more independent and rely less on supplementary support by family members.

The linear regression models revealed that the largest impact on burden scores was the hours of close supervision and the lack of completion of vision rehabilitation services. However, the relatively low *R* ^2^ values reveal that there are other potential covariates affecting their burden scores that our models did not account for. How caregivers could endorse greater sentiments of burden with increasing time spent providing close personal care is readily understandable.

Caregivers of patients who are legally blind do not seem to be more at risk for depression than caregivers of low vision patients. Although a larger number of participants may have revealed a statistically significant difference between the two groups, both values are fairly low. Again, we believe the widespread utilization of vision rehabilitation services among patients resulted in the acquisition of greater skills, tools, and coping mechanisms, which in turn translated to less reliance on caregivers. Our data reveals that caregivers of blind patients experience significantly more burden than caregivers of low vision patients. Yet this difference is not seen when it comes to being at risk for depression. It can be speculated that although burden and depression tend to correlate, there exists a spectrum of severity, whereby only high levels of burden may translate to concomitant depression. Since the overall burden in our sample was lower in severity, there was no meaningful difference regarding the proportion of caregivers at risk for depression.

Similar to the regression model for burden, the factors having the greatest impact on the risk of depression were hours of close supervision and the lack of completing vision rehabilitation services. An additional risk factor identified here was female gender among caregivers. Depression has been cited to be more common in females in many landmark studies [31, 32] and perhaps may not reveal anything new about the dynamic of providing care to visually impaired patients. Moreover, this may just be a reflection of our sample of caregivers being predominantly female (59%).

There are some noteworthy limitations in this study. First, we relied on convenience sampling among caregivers who agreed to be interviewed. The caregivers and patients refusing to do the survey may be systematically different from those who completed the study. Second, this cross-sectional design prevents any causal relationship to be implicated between blindness or low vision and caregiver burden or depression. Longitudinal investigations will help elucidate this relationship. Third, psychiatric histories for the caregivers were not obtained and may have been relevant predictors of depression. Caregivers with concurrent psychiatric comorbidities were not formally excluded and this may have also confounded our results. Fourth, we did not look at social problem-solving abilities which have been shown to be correlated with caregiver burden and depression [28], namely, a negative orientation to problem-solving (believing one cannot solve a problem no matter how hard they try), impulsive/careless outlook (proceeding with the first idea that comes to mind when trying to solve a difficult problem), and an avoidant outlook (procrastinating to solve problems that occur in one's life). Fifth, we did not have a comparison group in this study to assess if there were differences specific in this sample regarding burden and depression among caregivers of legally blind or low vision patients and similar caregivers of patients who were not legally blind or did not have low vision. However, it was unrealistic to obtain several hundred controls, given our stringent exclusion criteria for comorbidities.

The major implications of this study for visual health specialists in Canada are threefold: first, to be cognizant that caregivers of legally blind or low vision patients may be at risk for depression as well as burden and these disorders should be considered when assessing the low vision patients; second, to recognize the various risk factors mentioned in this study associated with either higher burden or risk of depression: greater daily hours of close supervision provided, patients not completing a vision rehabilitation program, and female gender among caregivers; third, the use of vision rehabilitation services not only provides skills to patients with low vision but will also mitigate sentiments of burden and depression amongst caregivers. Future directions of this study include introduction of additional surveys that provide insight into the overall quality of life of caregivers despite overall increases in care burden. One such survey is the Quality of Life questionnaire. Furthermore, the addition of questionnaires and scales such as the Cornell Depression Scale, the Hamilton Scale for Depression, or the Beck Depression Inventory could provide valuable clinical information that may assist physicians in the formulation of diagnoses of depressive disorders. They may also be valuable life-saving tools that enable the investigation of suicide ideation that would otherwise go unnoticed.

## Figures and Tables

**Table 1:** Caregiver and patient characteristics, *N* = 236.

Mean patient age in years ± SD	76.4 ± 12.1
Patient gender, *N* (%):	
Male	109 (46)
Female	127 (54)
Group by visual acuity, *N* (%):	
Group 2 (legally blind)	76 (32)
Group 1 (low vision, but not legally blind)	160 (68)
Completed vision rehabilitation in past, *N* (%):	
Yes	169 (72)
No	67 (28)
Etiologies of diminished vision, *N* (%)	
Age related macular degeneration	170 (72)
Secondary to diabetic disease	38 (16)
Prior vascular occlusion	28 (7)
Caregiver gender, *N* (%):	
Male	97 (41)
Female	139 (59)
Mean caregiver age, years ± SD	64.8 ± 10.2
Relationship with patient, *N* (%):	
Child	70 (30)
Spouse	122 (52)
Sibling	31 (13)
Other (friend/grandchild)	13 (6)
Caregivers with chronic illness, *N* (%):	
None	175 (74)
One or more chronic illnesses	61 (26)
Patients with chronic illness, *N* (%):	
None	126 (52)
One or more chronic illnesses	110 (48)
Duration of caregiving, years, mean ± SD	6.1 ± 5.6
Hours required for close supervision of the patient per day, mean ± SD	2.2 ± 0.7
Number of supplemental caregivers, mean ± SD	0.37 ± 0.68

BCVA: best corrected visual acuity. Group 1: best corrected visual acuity in the better eye > 6/60 yet worse than 6/18. Group 2: best corrected visual acuity in the better eye ≤6/60 (legally blind).

**Table 2:** The modified Burden Index of Caregivers scores among caregivers of patients with varying degrees of visual impairment.

	Group 1	Group 2	*P* value
Time-dependent burden	0.58 ± 0.37	2.12 ± 0.89	<0.01
Emotional burden	0.37 ± 0.24	1.54 ± 0.63	<0.01
Existential burden	0.20 ± 0.17	1.15 ± 0.52	<0.01
Physical burden	0.21 ± 0.13	1.22 ± 0.58	<0.01
Service-related burden	0.17 ± 0.27	0.95 ± 0.43	<0.01
Personal estimate of overall burden	0.20 ± 0.12	1.32 ± 0.71	<0.01
Total mean BIC	2.03 ± 0.63	8.01 ± 2.25	<0.01

*P* value calculated using independent two-tailed *t*-tests showed that values for Group 2 were significantly higher for each domain. Group 1: best corrected visual acuity in the better eye > 6/60 yet worse than 6/18. Group 2: best corrected visual acuity in the better eye ≤6/60 (legally blind).

**Table 3:** Covariates impacting caregiver burden (2 linear regression models).

	Regression coefficient	Standard error	*P* value
*Personal estimate of overall burden* (*R* ^2^ = 0.38)			
Hours of close supervision	0.67	0.34	<0.01
Completion of vision rehabilitation			
Yes (reference)	—	—	—
No	0.59	0.34	0.04
*Modified BIC total* (*R* ^2^ = 0.33)			
Hours of close supervision	0.94	0.31	<0.01
Completion of vision rehabilitation			
Yes (reference)	—	—	—
No	0.64	0.36	0.04

BIC: Burden Index of Caregivers.

**Table 4:** Covariates impacting the risk of depression among caregivers in the logistic regression model.

	Odds ratio (95% confidence intervals)	*P* value
Caregiver gender		
Male (reference)	—	
Female	5.39 (2.92–9.14)	<0.01
Completed vision rehabilitation		
Yes (reference)	—	
No	4.23 (1.32–7.32)	<0.01
Hours required for close supervision		
<2.5 hours (reference)	—	
≥2.5 hours	7.45 (3.45–10.34)	<0.01

**Table 5:** Which best describes how often you felt or behaved during the last week?

	Rarely or none of the time (less than 1 day)	Some or a little of the time (1-2 days)	Occasionally or a moderate amount of the time (3-4 days)	Most or all of the time (5–7 days)
During the past week:	0	1	2	3
(1) I was bothered by things that usually don't bother me	0	1	2	3
(2) I did not feel like eating; my appetite was poor	0	1	2	3
(3) I felt that I could not shake off the blues even with help from my family and friends	0	1	2	3
(4) I felt that I was just as good as other people	0	1	2	3
(5) I had trouble keeping my mind on what I was doing	0	1	2	3
(6) I felt depressed	0	1	2	3
(7) I felt that everything I did was an effort	0	1	2	3
(8) I felt hopeful about the future	0	1	2	3
(9) I thought my life had been a failure	0	1	2	3
(10) I felt fearful	0	1	2	3
(11) My sleep was restless	0	1	2	3
(12) I was happy	0	1	2	3
(13) I talked less than usual	0	1	2	3
(14) I felt lonely	0	1	2	3
(15) People were unfriendly	0	1	2	3
(16) I enjoyed life	0	1	2	3
(17) I had crying spells	0	1	2	3
(18) I felt sad	0	1	2	3
(19) I felt that people disliked me	0	1	2	3
(20) I could not get “going”	0	1	2	3

## Appendices

### A. Original Burden Index of Caregivers (BIC) Questionnaire

*Time-Dependent Burden*

1. I cannot freely leave the house because of care-giving
2. I do not have enough time for myself because of care-giving

*Emotional Burden*

(3) I want to delegate the care to someone else
(4) I am completely distressed by care-giving

*Existential Burden*

(5) I am experiencing hardship because care-giving does not give me a sense of satisfaction
(6) Care-giving is hard because I cannot find the meaning of providing care

*Physical Burden*

(7) My body aches when providing care to my family member
(8) I have ruined my health in the course of providing care

*Service-Related Burden*

(9) It is a burden that public aid service personnel enter our house
(10) I have a hard time because patients resent receiving public aid care services

*Personal Estimate of Overall Burden*

(11) How burdensome do you think providing care is to you?

*Legend*. Each question was rated on a Likert scale: 0, never; 1, almost never; 2, sometimes; 3, often; 4, always.

Adapted from [4].

### B. Center for Epidemiologic Studies Depression (CES-D) Scale

See Table 5.
